# Detection of Respiratory Events during Sleep Based on Fusion Analysis and Entropy Features of Cardiopulmonary Signals

**DOI:** 10.3390/e25060879

**Published:** 2023-05-30

**Authors:** Xinlei Yan, Juan Liu, Lin Wang, Shaochang Wang, Senlin Zhang, Yi Xin

**Affiliations:** 1 School of Medical Technology, Beijing Institute of Technology, Beijing 100081, China; 2 Jihua Laboratory, Foshan 528200, China; 3 School of Life Science, Beijing Institute of Technology, Beijing 100081, China

**Keywords:** machine learning, SAHS, apnea event, entropy, respiratory signal

## Abstract

Sleep apnea hypopnea syndrome (SAHS) is a common sleep disorder with a high prevalence. The apnea hypopnea index (AHI) is an important indicator used to diagnose the severity of SAHS disorders. The calculation of the AHI is based on the accurate identification of various types of sleep respiratory events. In this paper, we proposed an automatic detection algorithm for respiratory events during sleep. In addition to the accurate recognition of normal breathing, hypopnea and apnea events using heart rate variability (HRV), entropy and other manual features, we also presented a fusion of ribcage and abdomen movement data combined with the long short-term memory (LSTM) framework to achieve the distinction between obstructive and central apnea events. While only using electrocardiogram (ECG) features, the accuracy, precision, sensitivity, and F1 score of the XGBoost model are 0.877, 0.877, 0.876, and 0.876, respectively, demonstrating that it performs better than other models. Moreover, the accuracy, sensitivity, and F1 score of the LSTM model for detecting obstructive and central apnea events were 0.866, 0.867, and 0.866, respectively. The research results of this paper can be used for the automatic recognition of sleep respiratory events as well as AHI calculation of polysomnography (PSG), which provide a theoretical basis and algorithm references for out-of-hospital sleep monitoring.

## 1. Introduction

Undiagnosed and untreated sleep apnea hypopnea syndrome (SAHS) is a major health burden worldwide and is associated with several severe health consequences, such as dementia [1], hypertension [2], cardiovascular disease [3,4,5], and even sudden death. Apnea refers to the decrease in airflow amplitude by more than or equal to 90% compared with baseline and the duration is greater than or equal to 10 s. The apnea events can be classified as obstructive, central, or mixed events on the basis of the presence of respiratory effort of the abdomen and chest, and their incidence in cases is 84%, 0.4%, and 15%, respectively [6]. Different apnea events affect the health of people differently, with central apnea events often associated with heart-related diseases, while obstructive apnea is more relevant to upper airway narrowing and metabolic disorders. In clinical practices, the polysomnography (PSG)-based method is the golden standard for sleep-related breathing disorders diagnosis. However, it is costly, time-consuming and requires the monitoring of multi-lead physiological signals for one or two nights at the sleep center of a hospital, while the output requires manual annotation of respiratory events by a sleep specialist, which is labor-intensive, error-prone and susceptible to subjectivity, making it a complex and expensive process [7,8,9]. The apnea hypopnea index (AHI) can be calculated based on the frequency of these events throughout the night. According to the AHI, the severity of SHAS can be classified as none (AHI < 5), mild (5 ≤ AHI < 15), moderate (15 ≤ AHI < 30), or severe (AHI ≥ 30) [10].

Various researchers performed the automatic detection of respiratory events using multi-sensors data. Yang et al. [11] investigated the automatic recognition of respiratory events using depth video and audio of a patient recorded using a Microsoft Kinect camera during his/her sleep. The method achieved the identification of four sleep respiratory events, and the average accuracy of their model was 99.60%. McCloskey et al. [12] applied a convolutional neural network (CNN) to a 2D image wavelet spectrogram of the nasal signal, and then built up a classification model for normal breathing, obstructive apnea and hypopnea events (with a sensitivity of 79.7%). Urtnasan et al. [13] proposed a CNN-based deep learning architecture for the multiclass classification of respiratory events using single-lead ECG recordings, with a sensitivity of 87.0%. Rolon et al. [14] presented a structured dictionary learning approach in conjunction with MLP to discriminate apnea and hypopnea segments from pulse oximetry (SpO_2_) signals. Wenming et al. [15] introduced an automatic SAHS event detection method based on the LSTM network via nasal airway pressure and temperature signals, with the sensitivity for normal breathing, apnea, and hypopnea events being 81.3%, 87.8% and 75.5%, respectively. A novel portable device was presented, combined with a two-phase LSTM deep learning algorithm for automated event detection, by Steenkiste et al. [16]. Recently, some other studies based on the model fusion method were able to effectively improve the accuracy and sensitivity of respiratory event recognition. Almutairi et al. [8] utilized deep learning approaches to learn the patterns and features of ECG signals, and the results showed that the architecture of CNN with LSTM performed best compared to CNN-only models. Bozkurt et al. [17] presented a machine learning-based system that can detect apnea during sleep. In their work, features extracted from the ECG signals were inserted into the model integrating DT, k-nearest neighbor (KNN), and support vector machines (SVM), and the fusion model performed better than the single classifier.

Although many of the studies mentioned above improve the accuracy of respiratory event detection on machine learning, they still have some limitations. First of all, some methods require a high level of sleep environment and data processing equipment. Furthermore, most studies are usually devoted to the detection of abnormal events, and if these exist, they usually focus only on the distinction between normal breathing, hypopnea, and apnea. As far as we know, there are no studies that have comprehensively focused on the specific typology of sleep respiratory events. In this paper, an automatic recognition method for respiratory events during sleep is introduced. Our main contributions are: (1) our algorithm had a higher sensitivity of 87.4% compared to previous studies in distinguishing between normal breathing, hypopnea and apnea events. (2) A fusion of two-lead data combined with the LSTM framework was presented to achieve the distinction between obstructive and central apnea events. (3) Based on the above algorithm, this paper proposed an application strategy for the AHI calculation of SHAS patients.

The remainder of the paper is organized as follows: Section 2 discusses the database and the methodology employed in this paper; Section 3 and Section 4 describe the results of the research work and discuss the outcomes of the algorithm application; and Section 5 concludes the paper.

## 2. Materials and Methods

**Figure 1:**
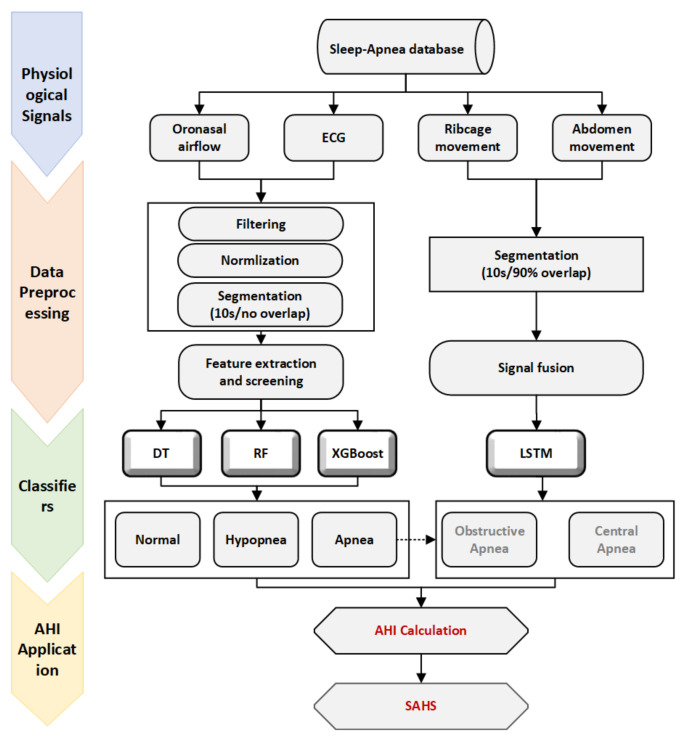
Overview of the proposed method for automatic detection of respiratory events.

The scheme of the proposed automatic respiratory event detection algorithm is depicted in Figure 1. First, we aimed to identify the three basic sleep respiratory events of normal breathing, hypopnea and apnea. As previously mentioned, there is a distinction between obstructive and central apnea events, so we incorporated the LSTM network architecture to further achieve a distinction between obstructive and central apnea events. Above all, we finally calculated the AHI index in order to distinguish the severity of SAHS patients.

### 2.1. Datasets

In this study, the Sleep-Apnea database on Physionet was used to develop the model [18,19]. This database contains 25 night-time recordings from 4 women and 21 men. The age of the subjects ranges from 28 to 68. The AHI index of the subjects ranges from 1.7 to 90.9. The lengths of the recordings are approximately 6–7.5 h. Each recording contains both the PSG overnight and the Holter ECG signal (the sampling frequency is 128 Hz) acquired simultaneously on three channels. The PSG was measured using the German Jaeger Toennies system, with the sampling frequency of airflow and the respiratory movement of abdomen and ribcage at 8 Hz. Respiratory events (Hypopnea, Obstructive, Central, Mixed) of recordings were labeled by professional physicists.

The sleep PSG database Sleep Heart Health Study (SHHS) was used to evaluate the effectiveness of our algorithm [20,21]. The entirety of the 5804 recordings were included, divided into SHHS1 (1995–1998) and SHHS2 (2001–2003) according to the time period of data collection. All subjects were aged 40 years and older. Only the ECG signal was used in this study as an input to verify the performance of the algorithm.

### 2.2. Preprocessing

In the pre-processing section, signal processing methods were used to remove noise and baseline drift from the original signal. For the oronasal airflow signal, a Butterworth lowpass filter with a 0.3 Hz cutoff frequency was considered. For the ECG signal, a median filter was used. Then, the oronasal airflow and ECG data of each subject were split into 10 s non-overlapping segments for the following steps. As the number of obstructive apnea and central apnea events was smaller, the slicing strategy in distinguishing between these two types of respiratory events became a 10 s window with an overlap rate of 90%. All these signal processing operations were performed with MATLAB (R2018b).

### 2.3. Feature Extraction

#### 2.3.1. Morphological Analysis

According to the definitions of respiratory events, the most significant difference between normal breathing, hypopnea, and apnea events lies in the amplitude variations of airflow. Therefore, we extracted morphological features such as respiratory frequency (RF), inspiratory duty cycle (IDC), inspiratory volume, expiratory volume, area under the inspiratory curve, area under the complete respiratory curve and the signal slope. The relevant morphological features are calculated as follows.

The RF is defined as the number of breaths per second, calculated as the reciprocal of the respiratory cycle, as shown in Equation (1).

(1) $$\mathrm{RF}=\frac{1}{\mathrm{Respiratory}\;\mathrm{Cycle}}$$

The IDC is defined as the ratio of the duration of inspiration to the duration of the complete respiratory process and is calculated as:

(2) $$\mathrm{IDC}=\frac{\mathrm{Inspiratory}\;\mathrm{Time}}{\mathrm{Respiratory}\;\mathrm{Cycle}}$$

The signal slope is defined as the slope of the signal during inspiration, calculated as the ratio of inspiratory volume to inspiratory time, as shown in Equation (3).

(3) $$\mathrm{Slope}=\frac{\mathrm{Inspiratory}\;\mathrm{Volume}}{\mathrm{Inspiratory}\;\mathrm{Time}}$$

#### 2.3.2. HRV Analysis

HRV is the fluctuation between consecutive heartbeat signals due to unstable dynamic balance within the cardiovascular system. The RR interval was extracted from the ECG signal using the Pan–Tompkins algorithm. The time domain features, frequency domain features, and nonlinear correlation features of HRV were analyzed. For the time-domain analysis, the following features were extracted: serial correlation coefficients, NN50 (the number of consecutive RR intervals differing at least 50 ms from each other), PNN50 (the ratio of NN50 to the total number of RR intervals), root mean square of deviation (RMSD), mean absolute deviation (MAD), coefficient of variation of RR intervals (CVRR) and triangular index. Features in the time domain can provide a preliminary description of the basic physiological signal, which can visualize the distribution, trend, and variability of the signal. For the frequency domain analysis, the following features were extracted: total power (<0.4 Hz), very low frequency, low frequency, normalized low frequency, high frequency, normalized high frequency, and the ratio of low frequency to high frequency. Frequency domain metrics can reflect sympathetic and parasympathetic nerve activity [22].

Nonlinear analysis enables one to characterize the orderliness, complexity and redundancy of time-series signals. In this study, the following six entropy features were adopted: Shannon entropy [23], Renyi entropy [24], Tsallis entropy [25], transfer entropy [26], Shannon entropy of the degree distribution [27] and alphabet entropy [28,29]. Another measure we adopted to visualize the nonlinear properties of RR interval series data is the Poincare plot. This is a graphical representation of the correlation between consecutive RR interval series which characterizes the current status of autonomic nervous system (ANS). The main features of the Poincare plot are SD1 (defined as the standard deviation of the projection on the identification line of the Poincare plot), SD2 (defined as the standard deviation of the projection of the Poincare plot on the line perpendicular to the identification line) and SD1/SD2.

#### 2.3.3. Cardiopulmonary Coupling Analysis

The cardiopulmonary coupling technology can measure the coupling between cardiac regulation and respiratory changes [30]. To realize the implementation of CPC technology, it is necessary to extract the RR interval signal and ECG-derived respiratory signal (EDR). In our study, the QRS slope range, which is defined as the difference between the maximum and the minimum slopes in the QRS complex (computed from the first derivative in a symmetric window of 100 ms centered around the R-wave), was used to extract the EDR signal. Then, we made three spline interpolations of the RR interval signal and the EDR signal and finally obtained sequences with a sampling rate of 4 Hz. Then, the $CPC\left(\omega \right)$ can be calculated as follows:

(4) $$CPC\left(\omega \right)=C_{xy}\left(\omega \right)\times S_{xy}{\left(\omega \right)}^{2}$$

where $C_{xy}\left(\omega \right)$ and $S_{xy}\left(\omega \right)$ are the coherence coefficient and mutual power spectrum of $x\left(t\right)$ and $y\left(t\right)$, respectively.

### 2.4. Signal Fusion Method

In this paper, we proposed a signal fusion technique based on two-lead signals, which is conducted through the following steps.

Step 1: The two-lead signals $x\left(n\right)=\left\{x_{1},\;x_{2},\ldots ,x_{N}\right\}$ and $y\left(n\right)=\left\{y_{1},{\;y}_{2},\ldots ,y_{N}\right\}$ are collected simultaneously from the physiological signal acquisition device, where N is the length of the signal.

Step 2: $x\left(n\right)$ and $y\left(n\right)$ are taken as the horizontal and vertical axes of the two-dimensional coordinate system, respectively, and the state points are defined as the points of each moment and corresponding coordinate values. Then, we link these state points in time order to form a state evolution track.

Step 3: For the state points of the evolution track, we find out the closest state points in turn and use their distances $d_{i}$ as a new time series *D*$=\left\{d_{1},{\;d}_{2},\ldots ,d_{N}\right\}$ to form a fusion signal. The distance between two points in this study is calculated as follows:

(5) $$d=\sqrt{{\left(x_{2}-x_{1}\right)}^{2}+{\left(y_{2}-y_{1}\right)}^{2}}$$

The fused signal keeps the rhythmic characteristics of the ECG signal, while the amplitude demonstrates a significant change.

### 2.5. Classification and Evaluation Metrics

The proposed method for the respiratory event recognition provided two outputs: the identification of abnormal breathing events and the subdivision of apnea events. Decision tree (DT), random forest (RF), and extreme gradient boosting (XGBoost) models were employed for the automatic identification of three sleep respiratory events (normal breathing, hypopnea, apnea). Precision, sensitivity, F1 score, and accuracy as well as ROC curve and AUC metrics are used to evaluate the model classification results. The performance of the LSTM in the training process was evaluated with the accuracy and loss function (loss) to further optimize the model parameters. In addition, the SHAP (SHapley Additive exPlanations) method was used in this research, which can provide desirable interpretations of the model performance and highlights the most important features for identifying respiratory events [31].

## 3. Results

### 3.1. Classification of Respiratory Events (Normal Breathing, Hypopnea, Apnea)

**Figure 2:**
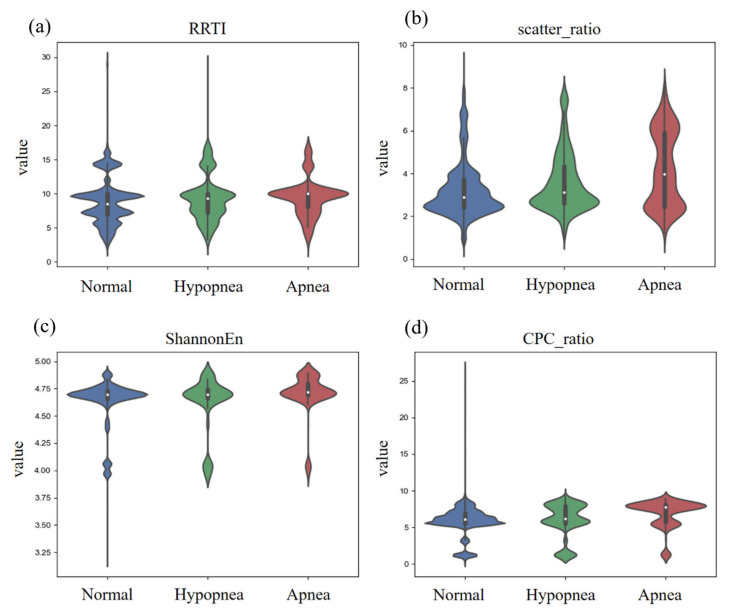
The distribution of some ECG-related features in different respiratory events. (**a**) Triangular index of RR interval. (**b**) SD1/SD2 parameters of RR interval scatter plot. (**c**) Shannon entropy based on RR interval. (**d**) Ratio of low and high frequency power from cardiopulmonary coupling analysis.

In this study, 52,896 normal breathing segments, 3436 hypopnea segments and 1019 apnea segments were finally obtained to select the most significant features and train the classification models. Based on the above feature selection methods, 46 features were significantly different. Figure 2 displays violin plots of four ECG-related features in different respiratory states. It can be seen that the features showed different distribution patterns in normal breathing, hypopnea and apnea events.

**Figure 3:**
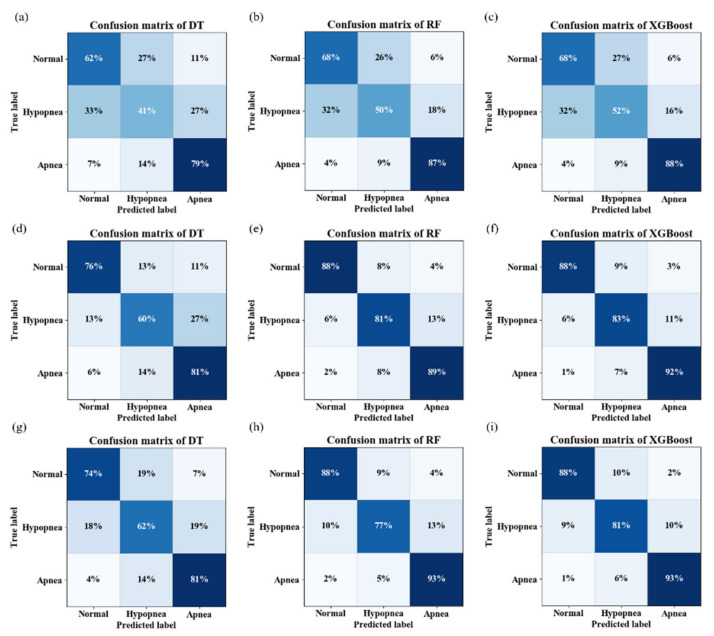
Confusion matrix of different classifiers. (**a**–**c**) Confusion matrix of DT, RF and XGBoost based on oronasal airflow-related features. (**d**–**f**) Confusion matrix of DT, RF and XGBoost based on ECG-related features. (**g**–**i**) Confusion matrix of DT, RF and XGBoost based on oronasal airflow and ECG features.

The results of the classification of the respiratory events for the Physionet Sleep-Apnea database are reported in Table 1. As seen in Table 1, when the input features were only from oronasal airflow, the XGBoost model had the best classification performance, with accuracy, precision, sensitivity, and F1 scores of 0.686, 0.681, 0.691, and 0.684, respectively. Moreover, the XGBoost classifiers can provide the best accuracy of 0.877, precision of 0.877, and sensitivity of 0.876, respectively, with the ECG features. When oronasal airflow and ECG features were used, the XGBoost algorithm also achieved high performance (ACC: 0.873, Sens: 0.874, and F1: 0.873). Figure 3 illustrated the confusion matrix of different classifiers. It can be seen from these figures that the sensitivity of normal breathing and apnea events classification was generally higher than that of hypopnea. In Figure 3i, the sensitivity of the XBGoost model for normal breathing, hypopnea, and apnea was 88%, 81%, and 93%, respectively.

**Figure 4:**
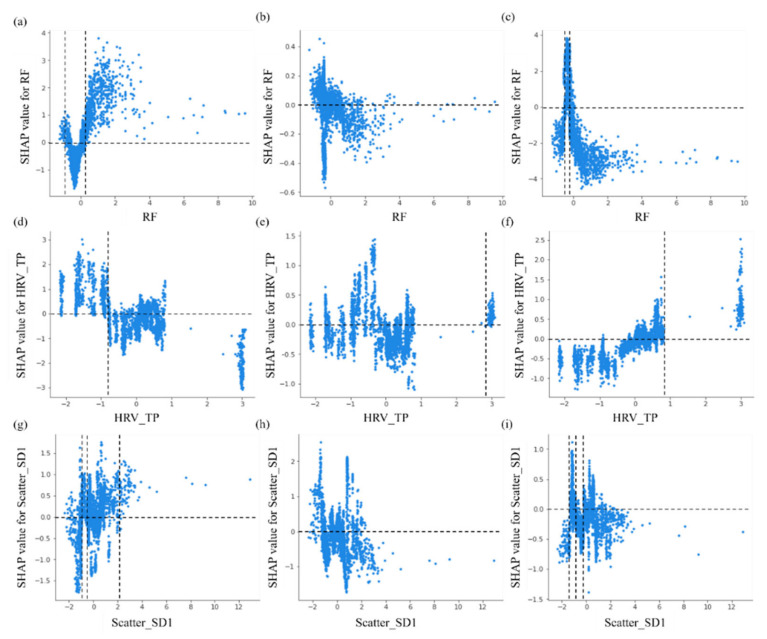
The impact of single features on the output of the XGBoost model. (**a**–**c**) The SHAP value for respiratory frequency for the conditions of normal breathing, hypopnea and apnea, respectively. (**d**–**f**) The SHAP value for the total power normalized in the frequency domain of the HRV analysis for the conditions of normal breathing, hypopnea and apnea, respectively. (**g**–**i**) The SHAP value for the SD1 parameter for the conditions of normal breathing, hypopnea and apnea, respectively.

The distribution of SHAP values for respiratory frequency, the total power of HRV analysis (HRV_TP) and the SD1 parameter are shown in Figure 4. From the results in Figure 4a–c, we notice that respiratory frequency is effective in identifying normal breathing from apnea events. The model tends to predict normal breathing when the normalized respiratory frequency is less than −1 or greater than 0.2 and outputs apnea events in the range of −0.4 to −0.2. Similarly, the values of −0.8 and 2.8 are the threshold values for differentiating normal breathing from hypopnea and apnea events based on the total power normalized in the frequency domain of the HRV analysis, as seen in Figure 4d–f. According to Figure 4g–i, it can be seen that the SHAP values of the SD1 parameter of the scatter plot of the RR interval are not clearly bounded in the three types of respiratory events and have a lower contribution to the output.

Table 2 shows classification results of XGBoost for each class of respiratory events with different numbers of features. In Table 2, it is reported that the first 10 features discriminated normal breathing events best with 89.6% precision, whereas for hypopnea event detection, the first 20 features were the best with 85.7% precision. Similarly, the classifier had the highest precision for detecting apnea events when the first 15 or 20 features were entered, both at 0.876.

Finally, 100 subjects were randomly selected from the SHHS1 database to evaluate our algorithm. The results of the classification of the events for the SHHS1 database are reported in Table 3. From Table 3, it can be seen that XGBoost classification for normal breathing events resulted in an accuracy of 0.930, sensitivity of 0.942, and F1 score of 0.936, while the sensitivity of the detection of hypopnea and apnea events was 0.877 and 0.902, respectively.

### 3.2. Classification of Apnea Events (Obstructive, Central)

**Figure 5:**
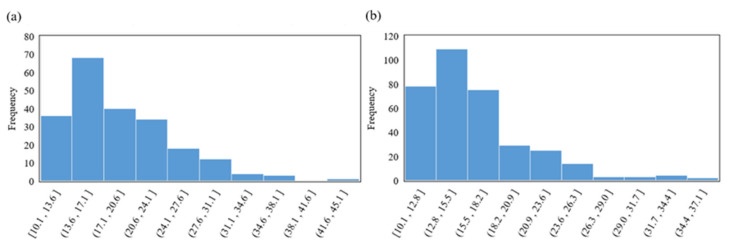
Frequency plots of the duration distribution. (**a**) Obstructive sleep apnea, (**b**) central sleep apnea.

**Figure 6:**
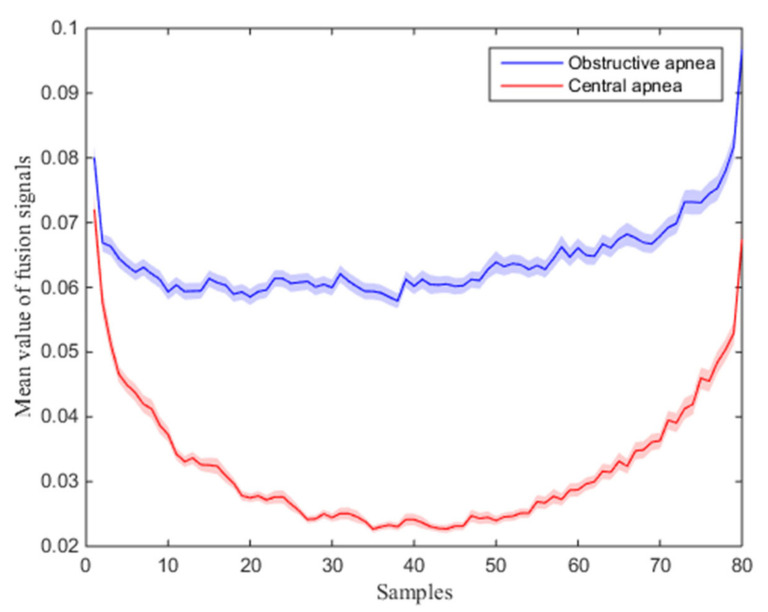
Distribution states of fusion signals in different respiratory events.

A total of 2171 obstructive apnea segments and 2405 central apnea segments were obtained in this study. Figure 5 shows the histogram of the frequency distribution of the duration of the obstructive and central apnea events. We derived the mean and standard error of the fusion signal of all obstructive and central apnea segments. Figure 6 demonstrates the distribution status of the fusion signal for different respiratory states. It can be seen that the mean amplitude of the fusion signal corresponding to obstructive apnea events is obviously higher than that of central apnea events.

**Figure 7:**
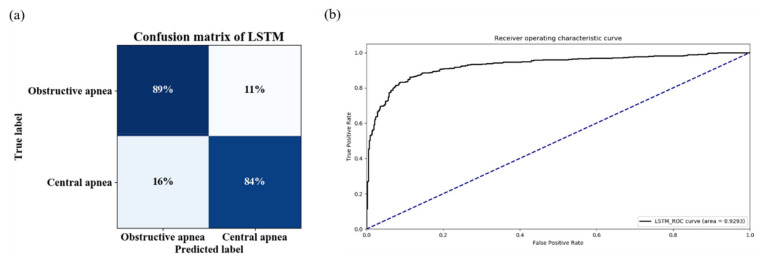
Confusion matrix and ROC curve for LSTM model. (**a**) Confusion matrix of the LSTM model. (**b**) ROC curves of the LSTM model.

As seen in Table 4, the accuracy, sensitivity and F1 score of the LSTM model for detecting obstructive apnea events were 0.832, 0.892, and 0.861, respectively. In the identification of central sleep apnea events, the LSTM results in terms of accuracy, sensitivity, and F1 score were 0.900, 0.843, and 0.870, respectively. The confusion matrix and ROC curve of this LSTM model are depicted in Figure 7. The value of AUC obtained was 0.9293, as illustrated in Figure 7b. Furthermore, we utilized ROC curve analysis to determine the optimal cutoff value (0.420) which was significant for identifying obstructive and central apnea events.

### 3.3. AHI Evaluation Framework

To verify the availability of the algorithm for the automatic recognition of sleep respiratory events based on ECG signals, 40 subjects were randomly selected from the SHHS1 database and individual AHI values were calculated based on the results of respiratory event prediction in this study. Table 5 shows the results of individual classification corresponding to different AHI value ranges. As shown, the precision of SAHS detection was 1.000 when the threshold value of AHI was set to 5, indicating the effectiveness of our algorithm for the screening of SAHS patients.

## 4. Discussion

The purpose of this study was to build an automatic recognition model of sleep respiratory events and explore the application scheme, so as to provide theoretical support for the automatic analysis of PSG data in the hospital, the preliminary screening of SAHS outside the hospital, and the real-time monitoring and alarm of apnea events. Almost all of the previous studies classify respiratory events based on single-channel ECG signals or airflow signals, without considering the synergistic effect. In this paper, we fused the relevant features of oronasal airflow signals and ECG signals and built a triple classification model of sleep respiratory events by using machine learning methods. As can be seen from the RF model, the sensitivity for apnea events would be 0.894 when only ECG-related features are input, but improved to 0.934 when oronasal airflow features are added. This implies that multi-signal features can add more information that contributes to building models for classification of respiratory events. The selection of the classifier also has an impact on the results. Compared to DT and RF, XGboost obtained the highest performance (accuracy of 87.3%, precision of 87.3%, and sensitivity of 87.4%). Based on the above results, it is clear that all the ECG and oronasal airflow features proposed in this study allow for high discrimination between normal breathing segments and different respiratory events. Our results provided a higher accuracy compared to Urtnasan et al. (2018), with a 0.7% increase in precision and 0.6% increase in sensitivity for the three classes problem. In this study, we have found that hypopnea events were always more difficult to detect than normal breathing and apnea events, and they were often classified as normal breathing. According to the American Academy of Sleep Medicine (AASM) definition of respiratory events, the biggest difference between these three types of respiratory events is the magnitude of the airflow, thus it is prone to misjudgment with regard to individual differences. For the optimal model XGBoost, we explored the interpretability of the model using the SHAP values. Thus, we could better understand the impact of features on the identification of each type of respiratory event by observing the distribution of SHAP values. The robustness of the proposed model was validated using 100 subjects from the SHHS1 database, and the XGBoost model also achieved high performance (accuracy of 93.0%, sensitivity of 94.2%, and F1 score of 93.6%). In addition, the specific classification of apnea events (obstructive and central) is often overlooked based on the literature. This is due to the fact that the precision and accuracy of the model decrease with the refinement of respiratory events. We propose fusing the two-lead thoracoabdominal motion signals into a one-dimensional time series, and then using LSTM to learn and train the temporal features of this fused signal. In the apnea events subclassification step, the proposed technique provided a precision of 86.6%, a sensitivity of 86.7%, and an F1 score of 86.6%.

To assess the feasibility of the method proposed in this study, we compared our detection method with other relevant works. In Table 6, the comparison between the results for the presented method and those of other existing algorithms in the classification of respiratory events is presented. As indicated in Table 6, the presented method along with the XGBoost classifier outperformed other relevant studies. Some studies such as that Choi et al. have achieved good performance, with an accuracy of 96.6%, precision of 87.0%, and sensitivity of 81.1%; however, the purpose of this study was only focused on the distinction between normal breathing and abnormal respiratory events [32]. The validation set we use is the same as the database employed by Steenkiste et al., but our method has a higher performance [33]. All in all, the proposed method in this study is better than other studies in this area in the classification of all three different respiratory events and apnea events subdivision.

Determining a patient’s sleep type generally requires labeling PSG data, which takes approximately 1–2 h for one subject, which makes PSG testing time-consuming and labor-intensive and subjective to the professional, allowing for some variation in the labeling of respiratory events. The automatic recognition of sleep events will greatly free up the labor of professional technicians, while the automatically labeled model can be embedded in clinically relevant devices to support use in a variety of scenarios, including home testing, extreme environments, special surgical conditions, and wearable devices. Based on the results obtained in this study, aimed to be embedded in clinical PSG testing terminals or everyday wearable devices, we also propose in this paper an automated assessment strategy for the AHI index, which enables the automated screening of SHAS patients out-of-hospital.

However, our study still has some limitations. On the one hand, our models are less effective in detecting hypopnea events than normal breathing and apnea events, and normal breathing is easily misclassified as a hypopnea event. On the other hand, the collected airflow signals may differ depending on the sensor, and different types of airflow signals may have different effects on the detection of respiratory events, which is left for future work.

## 5. Conclusions

In this article, we have presented an automatic recognition model for respiratory events during sleep based on the Sleep-Apnea database. In addition, a signal fusion method was proposed to extract new features for the discrimination of the different respiratory apnea events. For the subdivision of apnea events, LSTM was employed to learn and train the fused signals. In the classification of three respiratory events, the proposed method performance using the XGBoost model in terms of accuracy, precision, and sensitivity were 87.3%, 87.4%, and 87.4%, respectively. Moreover, the LSTM network with fused signals to distinguish obstructive and central apnea events yielded the results with an accuracy of 86.6%, precision of 86.7%, and sensitivity of 86.6%. The results indicate that our automatic algorithm allows for the application of a real-time sleep monitoring system and helps physicians to gain a comprehensive understanding of the sleep status of subjects.

## Figures and Tables

**Table 1:** The overall performances of different classifiers.

Feature Source	Metrics	DT	RF	XGBoost
Oronasal airflow	Pre	0.594	0.675	0.681
	Sen	0.608	0.684	0.691
	F1	0.597	0.677	0.684
	Acc	0.603	0.680	0.686
ECG	Pre	0.724	0.863	0.877
	Sen	0.720	0.862	0.876
	F1	0.720	0.862	0.876
	Acc	0.723	0.863	0.877
ECG + oronasal airflow	Pre	0.724	0.859	0.873
	Sen	0.727	0.860	0.874
	F1	0.725	0.858	0.873
	Acc	0.725	0.858	0.873

**Table 2:** Performance of XGBoost for each class of respiratory events with different number of feature inputs.

Class	Top10			Top15			Top20		
	Pre	Sen	F1	Pre	Sen	F1	Pre	Sen	F1
N	0.896	0.892	0.894	0.895	0.889	0.892	0.885	0.895	0.890
H	0.849	0.807	0.827	0.846	0.808	0.827	0.857	0.801	0.828
A	0.874	0.926	0.899	0.876	0.924	0.899	0.876	0.928	0.901

**Table 3:** Performance of XGBoost in SHHS1 database.

Class	Pre	Sen	F1	Acc
N	0.930	0.942	0.936	0.907
H	0.881	0.877	0.879	
A	0.910	0.902	0.906	
Average	0.907	0.907	0.907	

**Table 4:** Performance of LSTM in apnea events detection.

Class	Pre	Sen	F1
Obstructive apnea	0.832	0.892	0.861
Central apnea	0.900	0.843	0.870
Average	0.866	0.867	0.866

**Table 5:** Model performance corresponding to different AHI thresholds.

AHI Threshold		Pre	Sen	F1	Number of Samples	Acc
5	AHI < 5	0.556	1.000	0.714	10	0.800
	AHI ≥ 5	1.000	0.733	0.846	30	
	Average	0.778	0.867	0.780	-	
15	AHI < 15	0.897	0.929	0.912	28	0.875
	AHI ≥ 15	0.818	0.750	0.783	12	
	Average	0.857	0.839	0.847	-	
30	AHI < 30	0.943	0.943	0.943	35	0.900
	AHI ≥ 30	0.600	0.600	0.600	5	
	Average	0.771	0.771	0.771	-	

**Table 6:** Comparison of respiratory events classification results between the proposed method and some existing algorithms.

Researchers	Number of Subjects	Signal Type	Model	Class	Results (%)			
					Acc	Pre	Sen	F1
Koley and Dey et al. (2013) [34]	28	Oronasal airflow	SVM	N/AH	89.8	90.0	93.2	-
Choi et al. (2018) [32]	129	Nasal pressure	CNN1D	N/AH	96.6	87.0	81.1	-
McCloskey et al. (2018) [12]	1507	Nasal airflow	CNN2D	N/H/A	-	79.8	79.7	79.7
Steenkiste et al. (2019) [33]	100	Abdominal respiratory	LSTM	N/A	77.2	39.9	62.3	-
ElMoaqet et al. (2020) [35]	17	Nasal pressure	BiLSTM	N/A	85.0	58.8	90.3	71.2
Urtnasan et al. (2018) [36]	82	ECG	CNN	N/A	-	96.0	96.0	96.0
Urtnasan et al. (2018) [13]	86	ECG	CNN	N/H/A	-	87.0	87.0	87.0
Our work	25	ECG + oronasal airflow	XGBoost	N/H/A	87.3	87.4	87.4	87.3
		Ribcage + abdomen movements	LSTM	OA/CA	86.6	86.6	86.7	86.6

SVM: support vector machine; CNN: convolutional neural network; LSTM: long short-term memory network; kNN: k-nearest neighbor; GRU: gated recurrent unit; LRM: logistic regression model; DT: decision tree; GMM: Gaussian mixture model; N: normal breathing; H: hypopnea event; A: apnea event; OA: obstructive apnea event; CA: central respiratory apnea events; MA: mixed apnea events; Acc: accuracy; Pre: precision; Sen: sensitivity; F1:F1 score.

## Data Availability

The data that support the findings of this study are available in https://physionet.org/ (accessed on 1 May 2022) and https://sleepdata.org/datasets (accessed on 1 May 2022).
